# Dietary protein intake and renal function

**DOI:** 10.1186/1743-7075-2-25

**Published:** 2005-09-20

**Authors:** William F Martin, Lawrence E Armstrong, Nancy R Rodriguez

**Affiliations:** 1 Department of Nutritional Sciences, University of Connecticut, Storrs, CT, USA; 2 Department of Kinesiology, University of Connecticut, Storrs, CT, USA

## Abstract

Recent trends in weight loss diets have led to a substantial increase in protein intake by individuals. As a result, the safety of habitually consuming dietary protein in excess of recommended intakes has been questioned. In particular, there is concern that high protein intake may promote renal damage by chronically increasing glomerular pressure and hyperfiltration. There is, however, a serious question as to whether there is significant evidence to support this relationship in healthy individuals. In fact, some studies suggest that hyperfiltration, the purported mechanism for renal damage, is a normal adaptative mechanism that occurs in response to several physiological conditions. This paper reviews the available evidence that increased dietary protein intake is a health concern in terms of the potential to initiate or promote renal disease. While protein restriction may be appropriate for treatment of existing kidney disease, we find no significant evidence for a detrimental effect of high protein intakes on kidney function in healthy persons after centuries of a high protein Western diet.

## Dietary protein intake and renal function

Dietary protein intake can modulate renal function [1] and its role in renal disease has spawned an ongoing debate in the literature. At the center of the controversy is the concern that habitual consumption of dietary protein in excess of recommended amounts promotes chronic renal disease through increased glomerular pressure and hyperfiltration [2,3]. Media releases often conclude that, "too much protein stresses the kidney" [4]. The real question, however, is whether research in healthy individuals supports this notion. In fact, studies suggest that hyperfiltration in response to various physiological stimuli is a normal adaptative mechanism [5-10].

The purpose of this paper is to review the available evidence regarding the effects of protein intake on renal function with particular emphasis on renal disease. This review will consider research regarding the role of dietary protein in chronic kidney disease, normal renal function and kidney stone formation and evaluate the collective body of literature to ascertain whether habitual consumption of dietary protein in excess of what is recommended warrants a health concern in terms of the initiation and promotion of renal disease. In the following review, high protein (HP) diets will be defined as a daily consumption of greater than or equal to 1.5 g/kg/day, which is almost twice the current Recommended Dietary Allowance but within the range of current Dietary Reference Intakes (DRIs) for protein [11]. The *Institute of Medicine *DRI report concluded that there was insufficient scientific evidence for recommendations of an upper limit of protein intake but suggested an acceptable macronutrient distribution range of 10–35% of total energy for protein intake [11].

While the optimal ratio of macronutrient intake for adults has typically focused on fat and carbohydrate [12], contemporary discussions include the role of dietary protein [13-15]. This is particularly true given the recent popularity of high protein diets in weight management [16]. Although the efficacy of these diets with regard to weight loss is still subject to debate, several studies have demonstrated favorable physiological effects [12,16-24]. This has led to a substantial increase in protein intake by individuals adhering to contemporary weight loss plans. As a result, the safety of habitually consuming dietary protein in excess of the Recommended Daily Allowance (RDA) has been questioned.

## An overview of chronic kidney disease

Chronic Kidney Disease (CKD) is defined as either kidney damage or a decline in renal function as determined by decreased glomerular filtration rate (GFR) for three or more months [25]. It is estimated that 1 in 9 adults in the United States meet this criteria, while an additional 1 in 9 adults are at increased risk for CKD [26]. In the general population, a decline in renal function is considered an independent risk factor for both cardiovascular disease and all-cause mortality [27]. However, the extent to which a mild diminution in renal function influences this risk is not known [28].

According to the National Kidney Foundation guidelines, CKD is classified into five stages, each of which directly correlates with the severity of the disease [25]. As one progresses from stage 1 to 5 there is a concomitant decline in GFR and thus renal function. The final stage, known as end stage renal disease, represents the most severe manifestation of CKD [29]. This classification system provides a universal standard for application of clinical treatment guidelines.

Hypertension is the second leading cause of CKD and accounts for approximately 30% of all cases in the U.S. [30,31]. In one study, hypertension was associated with a premature decline in renal function in men with normal kidney function [32]. Although, initial estimates of CKD prevalence in hypertensive individuals were about 2%, recent evidence suggests that prevalence rates may be significantly higher [33]. Blood pressure control is of particular importance in hypertensive individuals with CKD. This point has been demonstrated in several trials in which antihypertensive therapy slowed the progression of CKD [34-36].

Race, gender, age and family history are four risk factors for CKD [37-40]. Recent findings suggest that modifiable lifestyle risk factors (i.e., physical inactivity, smoking, obesity) are also associated with CKD. Limited data exist regarding the role of dietary protein intake as an independent risk factor for either the initiation or progression of renal disease but population studies have consistently demonstrated an inverse relationship between dietary protein intake and systemic blood pressure [41,42]. In a randomized control trial [43], dietary protein and fiber had additive effects in lowering 24-hour and awake systolic blood pressure in a group of 36 hypertensives. While these findings suggest that high protein diets may be beneficial to hypertensive individuals, additional research is warranted since increased protein intakes often result in increased consumption of certain micronutrients known to impact blood pressure (e.g., potassium, magnesium, calcium) [44].

## Dietary protein and renal function

The relationship between dietary protein and renal function has been studied for over half a century [1]. In 1923, Addis and Drury [45] were among the first to observe a relationship between level of dietary protein and rates of urea excretion. Soon after, it was established that increased protein intake elevated rates of creatinine and urea excretion in the dog model [46]. The common mechanism underlying increased excretion rates was eventually attributed to changes in GFR [47,48] and Van Slyke et al. [49] demonstrated that renal blood flow was the basis for GFR mediated changes in clearance rates in response to increased protein intake. Clearly dietary protein effects GFR [50], with both acute and chronic increases in protein consumption elevating GFR [50,51].

## Dietary protein and the progression of renal disease

**Figure 1:**
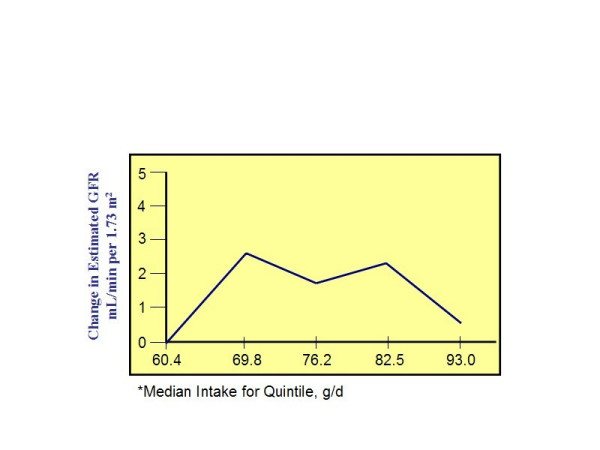
This figure is a plot of multivariate linear regression for change in estimated GFR according to quintile of total protein intake* in participants with normal renal function (n = 1135). Data are taken from Knight et al., *Ann Intern Med *2003 Mar 18;138(6):460-7 [53].

Observational data from epidemiological studies provide evidence that dietary protein intake may be related to the progression of renal disease [52]. In the Nurses' Health Study, protein intake, assessed with a semi-quantitative food frequency questionnaire, was compared to the change in estimated GFR over an 11-year span in individuals with *pre-existing *renal disease [53]. Regression analysis showed an association between increased consumption of animal protein and a decline in renal function suggesting that high total protein intake may accelerate renal disease leading to a progressive loss of renal capacity. However, no association between protein intake and change in GFR was found in a different cohort of 1,135 women with *normal *renal function (Figure 1.). The latter finding led the authors to conclude that there were no adverse effects of high protein intakes on kidney function in healthy women with normal renal status.

Research by Johnson et al. [54], showed protein intake as a possible risk factor for progressive loss of remaining renal function in dialysis patients. Indeed, dietary protein restriction is a common treatment modality for patients with renal disease [55,56] and practice guidelines exist regarding reduced dietary protein intakes for individuals with chronic renal disease in which proteinuria is present [57]. The National Kidney Foundation (NKF) has extensive recommendations with regard to protein intake, which are a byproduct of the Dialysis Outcome Quality Initiative [58]. Again, it is important to note that these recommendations are not indicated for individuals with normal renal function nor are they intended to serve as a prevention strategy to avoid developing CKD. Despite the clarity of these guidelines, their mere existence has resulted in concern regarding the role of dietary protein in the onset or progression of renal disease in the general population [59].

## Dietary protein and renal disease

Allen and Cope's observation that increased dietary protein induced renal hypertrophy in dogs [60] led to speculation that dietary protein intake may have deleterious effects on the kidney. Later research in the rat model produced evidence supporting earlier observations from canine research [61-63]. Recently, Hammond and Janes [64] demonstrated an independent effect of increased protein intake on renal hypertrophy in mice. In this study, changes in renal function (i.e., increased glomerular filtration rate and renal hypertrophy) were observed.

Currently, a combination of hormonal interactions and renal processes are thought to explain protein-induced hyperfiltration [65]. Increased glucagon secretion in response to protein administration induced hyperfiltration [66] subsequent to a cascade of events referred to as the"pancreato-hepatorenal cascade" [67]. It has been hypothesized that cAMP works in concert with glucagon to mediate GFR [68]. To date, however, this hypothesis has not been tested and other competing hypotheses suggest other novel mechanisms of protein-induced hyperfiltration [69].

While the effect of hyperfiltration on renal function in those individuals with pre-existing renal disease is well documented [52], the application of these observations to healthy persons with normal renal function is not appropriate. To date, scientific data linking protein-induced renal hypertrophy or hyperfiltration to the initiation or progression of renal disease in healthy individuals is lacking. The possibility that protein-induced changes in renal function are a normal physiological adaptation to nitrogen load and increased demands for renal clearance is supported by changes noted in renal structure and function during pregnancy [70]. GFR increases by as much as 65% in healthy women [8] during pregnancy, typically returning to nonpregnant levels by three months postpartum [7]. Despite these changes in renal function, pregnancy is not a risk factor for developing CKD [6].

The renal hypertrophy and accompanying improvements in renal function in the contralateral kidney that occur subsequent to unilateral nephrectomy also suggest these processes are an adaptive, and possibly beneficial, response [5]. Studies show, despite prolonged hyperfiltration, remnant kidney function remained normal and did not deteriorate during long-term (> 20 yrs) follow-up in nephrectomized patients [9,10]. Thus, compensatory hyperfiltration appears to be a biological adaptation to a variety of renal challenges that is not associated with increased risk of chronic kidney disease in healthy individuals.

## The Brenner Hypothesis

Perhaps the most consistently cited reference with regard to the potentially harmful effects of dietary protein intake on renal function is that of Brenner et al. [3]. In brief, the Brenner Hypothesis states that situations associated with increased glomerular filtration and glomerular pressure cause renal injury, ultimately compromise renal function, and potentially increase the risk for or progression of renal disease. Brenner proposed that habitual consumption of excessive dietary protein negatively impacted kidney function by a sustained increased in glomerular pressure and renal hyperfiltration [3]. Since the majority of scientific evidence cited by the authors was generated from animal models and patients with co-existing renal disease, extension of this relationship to healthy individuals with normal renal function is inappropriate. Indeed, a relationship between increased glomerular pressure or hyperfiltration and the onset or progression of renal disease in healthy individuals has not been clearly documented in the scientific literature. Rather, findings from individuals with compensatory hyperfiltration during pregnancy and following unilateral nephrectomy suggest otherwise [9].

The *Modification of Diet in Renal Disease *(MDRD) study was the largest randomized multicenter, controlled trial undertaken to evaluate the effect of dietary protein restriction on the progression of renal disease [71]. Several variables, including GFR, were measured in patients with chronic renal disease at baseline and throughout the approximately 2 year follow-up period. Patients with renal disease randomized to the very low-protein diet group had slightly slower decline in GFR decline compared with patients randomized to the low-protein diet group. Further data analyses showed patients with lower total protein intake would have a longer time to renal failure and suggested that a lower protein intake postponed the progression of advanced renal disease. Using meta-analysis to assess the efficacy of dietary protein restriction in previously published studies of diabetic and nondiabetic renal diseases, including the MDRD Study, Pedrini et al. concluded that the progression of both nondiabetic and diabetic renal disease could be effectively delayed with restriction of dietary protein [56]. Indeed, current clinical guidelines for the management of patients with renal disease continue to be based on the premise that protein intake greater than that recommended or which results in a renal solute load in excess of the kidney's excretory capabilities will contribute to progressive renal failure in persons with compromised renal function. However, of significance to this review, is the fact that imposing these guidelines on healthy individuals with normal renal function is overzealous given the current status of the scientific literature in this area.

## Dietary protein and renal strain

Concerns about level of dietary protein and renal function are often presented in public health guidelines [59]. In addition to the claims that high protein intake causes renal disease, some studies have suggested that renal function may be negatively affected by routine consumption of high protein diets [72-75]. Although high protein diets cause changes in renal function (i.e., increased GFR) and several related endocrine factors [1,76,77] that may be harmful to individuals with renal disease [52,53], there is not sufficient research to extend these findings to healthy individuals with normal renal function at this time.

The lay public is often told that high protein diets "overwork" the kidney and may negatively impact renal function over time [78]. In addition, a number of highly regarded organizations appear to support this line of reasoning [79] given the physiological processes required for excretion of protein-related metabolic waste products to maintain homeostasis following consumption of protein at levels in excess of recommended amounts. Increased consumption of dietary protein is linearly related to the production of urea [80] and urea excretion is controlled by the kidney. These processes are of significant energetic cost to the kidney and represent the physiological "strain" associated with increased protein intake [81].

The word "strain" is misleading given its negative connotation. In a press release [82], one group asserted that increased dietary protein "strains" the kidney via increased urea production, and causes dehydration and accumulation of blood urea nitrogen. This press release also suggested that these events synergistically overwork the kidney and predispose humans to CKD. Scientific research is often misrepresented in this context. Research from our laboratory [83] which is cited in the press release, does not support these contentions. Rather, we found that habitual consumption of a high protein diet minimally affected hydration indices. Changes in total body water and renal function were not measured.

The concept that increased dietary protein leads to dehydration may have originated from an unsubstantiated extension of a 1954 review of the nitrogen balance literature [84]. This review focused on the design of survival rations for military operations in the desert or at sea, when water supply and energy intake are limited. Since the excretion of 1 gram of urea nitrogen requires 40 – 60 mL of additional water, increased protein intakes in the study translated into an increased water requirement (i.e., +250 mL water per 6 grams of dietary nitrogen in a 500 Kcal diet) for excretion of urea nitrogen. This increased fluid requirement is situation specific and is not necessarily applicable to individuals whose calorie and water intakes are adequate. Presently, we know of no studies executed in healthy individuals with normal renal function which demonstrate a clear relation between increased dietary protein intake and dehydration or a detrimental "strain" on the kidney. Therefore, claims that a high protein diet promotes dehydration or adversely "strains" the kidney remain speculative.

## Evidence in healthy individuals

Although the efficacy of high protein diets for weight loss has been evaluated, there have been no reports of protein-induced diminutions in renal function despite subject populations that are generally at risk for kidney disease (e.g., dyslipidemia, obesity, hypertension) [14,15,22,85-87]. A randomized comparison of the effects of high and low protein diets on renal function in obese individuals suggested that high protein diets did not present a health concern with regard to renal function their study population [65]. In this study, 65 overweight, but otherwise healthy, subjects adhered to a low or high protein diet for six months. In the high protein group, both kidney size and GFR were significantly increased from that measured at baseline. No changes in albumin excretion were noted for either group and the authors concluded that, despite acute changes in renal function and size, high protein intake did not have detrimental effects on renal function in healthy individuals. Similar findings were recently reported by Boden et al. [88] in a study of 10 subjects who consumed their typical diet for 7 days followed by strict adherence to a high protein diet for 14 days. No significant changes were noted in serum or urinary creatinine and albumin excretion, suggesting no ill-effects of a high protein diet on renal function.

Athletes, particularly in sports requiring strength and power, consume high levels of dietary protein [89,90]. In fact, many athletes habitually consume protein in excess of 2.0 g/kg/day [91]. Supplementation with amino acids will further increase dietary protein levels in these individuals [92]. Yet there is no evidence that this population is at greater risk for kidney disease or losses in renal function [90]. Poortsmans and Dellalieux [93] found that protein intakes in the range of ~1.4–1.9 g/kg/day or 170–243% of the recommended dietary allowance did not impair renal function in a group of 37 athletes. We found no data in the scientific literature to link high protein intakes to increased risk for impaired kidney function in healthy, physically active men and women.

## Dietary protein and renal function in animal models

Although there is limited research regarding the long-term effects of high protein intakes on renal function in humans, animal models have provided insight into this quandary. Mammals fed acute and chronic high protein diets exhibit increases in GFR and renal blood flow [94]. These changes, which are comparable to those observed in humans, led to the hypothesis that high protein intakes are associated with progressive glomerulosclerosis in the rat. Recently, Lacroix et al. [95] studied the effects of a diet containing 50% protein on renal function in Wistar rats and noted no abnormalities in renal function or pathology. Collins et al. [96] also reported no adverse effects of long-term consumption of high protein diets on renal function when two years of a diet containing 60% protein failed to evoke changes in the percentage of sclerotic glomeruli in rats. Robertson et al., [97] studied the effect of increased protein intake on hyperperfusion and the progression of glomerulosclerosis in dogs that were 75% nephrectomized. After four years of feeding diets that were either 56, 27 or 19% protein, no association between diet and structural changes in the kidney were observed.

To the best of our knowledge, there has been only one report of a potentially toxic effect of excessive protein intake on renal function in the rat. Stonard et al. [98] found a diet containing 33% protein produced tubular damage in a specific strain of female rats. However, findings from this study are limited by the fact that damage was induced by a bacterial single-cell protein (Pruteen).

In summary, studies documenting high protein intake as a cause of renal disease in any animal model have not been done. Rather, studies have typically focused on the interaction between protein intake and renal function in the diseased state. As a result, findings from these investigations should not be used as a basis for dietary recommendations for humans. Studies designed to characterize the effects of dietary protein intake on renal function in healthy subjects are warranted.

## Dietary protein and kidney stones

The role of high protein diets in kidney stone formation has received considerable attention. Excessive protein intake increases excretion of potentially lithogenic substances such as calcium and uric acid [99,100]. Reddy et al. [101] noted that consumption of a high protein diet for six weeks was associated aciduria and urinary calcium and claimed that this constituted increased risk of stone formation in ten healthy subjects although none of the ten subjects developed renal stones. The severe carbohydrate restriction imposed in this study may have increased keto-acid production thereby contributing acid formation. Since consumption of fruits and vegetables usually produces a marked base load [102], restriction of these foods subsequent to the diet intervention may have also contributed to the net acid load.

Studies that claim an increased propensity for stone formation as a result of increased protein intake should be taken at face value because propensity is a surrogate marker and does not represent actual stone formation. Further, randomized control trials have not been done to test whether an increased tendency for stone formation is enhanced with consumption of a high protein diet.

Epidemiological studies provide conflicting evidence with regard to the association between protein intake and the predisposition for kidney stone formation. In a prospective study of over 45,000 men, researchers found a direct correlation between animal protein intake and risk of stone formation [103]. However, findings in women are difficult to interpret due to conflicting reports in the literature. While some studies have shown a direct relationship between animal protein intake and risk of stone formation in women [104,105], other work suggests an inverse relationship exists [106].

Conflicting findings regarding the role of dietary protein in kidney stone formation limit the development of universal guidelines with regard to a recommended protein intake for individuals at increased risk for stone formation [107]. It is not likely that diet alone causes kidney stone formation [108]. Rather, metabolic abnormalities are typically the underlying cause [109]. For example, Nguyen et al. [110] found that high intakes of animal protein adversely affected markers of stone formation in those afflicted with a stone causing disorder, while no changes were observed in healthy individuals. It has been suggested that one must have a preexisting metabolic dysfunction before dietary protein can exert an effect relative to stone formation [108]. This notion has been coined the "powderkeg and tinderbox" theory of renal stone disease by Jaeger [111]. This theory asserts that dietary excesses, such as high protein intake, serve as a tinderbox which, only in tandem with a metabolic abnormality (the powderkeg), can bring about stone formation. At the present time, however, evidence showing that a high protein intake is an inherent cause of this renal abnormality or is consistently associated with increased kidney stone formation does not exist.

## Conclusion

Although excessive protein intake remains a health concern in individuals with pre-existing renal disease, the literature lacks significant research demonstrating a link between protein intake and the initiation or progression of renal disease in healthy individuals. More importantly, evidence suggests that protein-induced changes in renal function are likely a normal adaptative mechanism well within the functional limits of a healthy kidney. Without question, long-term studies are needed to clarify the scant evidence currently available regarding this relationship. At present, there is not sufficient proof to warrant public health directives aimed at restricting dietary protein intake in healthy adults for the purpose of preserving renal function.

## Competing interests

The author(s) declare that they have no competing interests.

## Authors' contributions

WFM conducted literature search, prepared the manuscript and assisted in presentation of final draft, LEA and NRR conceived the idea, organized contents and participated in preparation of final manuscript.
